# Bone turnover markers predict changes in bone mineral density in men treated with abaloparatide: results from the abaloparatide for the treatment of men with osteoporosis (ATOM) study

**DOI:** 10.1093/jbmr/zjaf003

**Published:** 2025-01-10

**Authors:** Richard Eastell, Jacques P Brown, Robert A Adler, E Michael Lewiecki, Neil Binkley, Eric S Orwoll, David Kendler, Bruce H Mitlak, Yamei Wang

**Affiliations:** Department of Clinical Medicine, School of Medicine and Population Health, University of Sheffield, Sheffield S10 2RX, South Yorkshire, United Kingdom; Department of Medicine, Centre de recherche du CHU de Québec, Laval University, Quebec, QC G1V 0A6, Canada; Endocrinology Section, Richmond Veterans Affairs Medical Center and Endocrine Division, Virginia Commonwealth University School of Medicine, Richmond, VA 23298, United States; Division of Metabolic Bone Diseases, University of New Mexico Health Sciences Center and New Mexico Clinical Research & Osteoporosis Center, Albuquerque, NM 87106, United States; Osteoporosis Clinical Research Program, University of Wisconsin Osteoporosis Clinical Research Program, Madison, WI 53705, United States; Department of Medicine, Oregon Health & Science University, Portland, OR 97239, United States; Department of Medicine, University of British Columbia, Vancouver, BC V6T 1Z4, Canada; Clinical Development, Radius Health Inc., Boston, MA 02210, United States; Biostatistics, Radius Health Inc., Boston, MA 02210, United States

**Keywords:** uncoupling index, bone turnover markers, PINP, CTX, abaloparatide, osteoporosis

## Abstract

Early increases in bone turnover markers (BTMs) in response to anabolic therapy correlate with 18-mo BMD increases in postmenopausal women with osteoporosis; however, this relationship has not been assessed in men. In this analysis, the correlation between changes from baseline in fasting intact serum procollagen type I N propeptide (PINP) and serum CTX at 1, 3, 6, and 12 mo and percent increase from baseline in BMD at 12 mo in men from the randomized phase 3 ATOM study (NCT03512262) were evaluated using Pearson’s correlation coefficients. The uncoupling index (UI), a measure of the balance between markers of bone formation (PINP) and bone resorption (CTX), with positive UI favoring bone formation, was calculated. Results in men were compared to 12-mo results for women from the ACTIVE study using the *z* score test after Fisher’s *Z* transformation. In abaloparatide-treated men, PINP increases at 1 mo (*r* = 0.485), 3 mo (*r* = 0.614), 6 mo (*r* = 0.632), and 12 mo (*r* = 0.521) were highly correlated (*p* < .0001) with 12-mo LS BMD increases. The mean UI for abaloparatide-treated men was greater than placebo as early as 1 mo (2.26 vs −0.25). At month 3, the mean UI for men was greater (1.32) than for women (0.88) (*p* < .001). There was a significant correlation between 3-mo UI and LS BMD at 12 mo in both men (*r* = 0.453; *p* < .001) and women (*r* = 0.252; *p* < .01). UI at months 6 and 12 were also significantly correlated with 12-mo LS BMD in men and women, but the correlation was stronger in men than women. These data support that early changes in BTMs in men treated with abaloparatide are associated with subsequent changes in BMD similar to what has been reported in women.

## Graphical Abstract

**Graphical Abstract:**
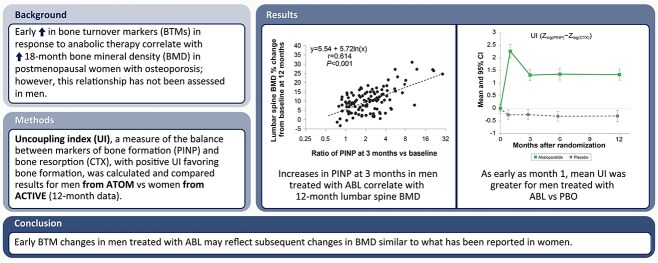

## Introduction

Osteoporosis in men remains underappreciated, underdiagnosed, and undertreated.^1^ Despite a lower prevalence of osteoporosis in men than in women, men have greater fracture-related morbidity and mortality.^2^^,^^3^ Approximately 25% of men over 50 yr of age will experience a fragility fracture in their lifetime, and men account for up to 30% of the societal burden of osteoporosis and fractures.^4^

Abaloparatide, a synthetic peptide analog of the human parathyroid hormone-related protein, favors bone formation by selective activation of parathyroid hormone receptor type 1.^5^ In the Abaloparatide for the Treatment of Men with Osteoporosis (ATOM; Clinicaltrials.gov identifier NCT03512262) study, abaloparatide treatment for 12 mo resulted in significant and rapid increases in BMD at the LS, TP, and FN compared with placebo in men with osteoporosis.^6^ Abaloparatide treatment also resulted in statistically significant changes in bone turnover markers (BTMs) consistent with the observed BMD increases. Median intact serum procollagen type I N propeptide (PINP) peaked at month 1, and serum CTX peaked at month 6. The increases in BTMs relative to baseline were significantly greater at all time points compared to placebo (*p* < .0001).^6^

The results of ATOM were consistent with the Abaloparatide Comparator Trial in Vertebral Endpoints (ACTIVE) study (Clinicaltrials.gov identifier NCT01343004), in which abaloparatide increased BMD in women with postmenopausal osteoporosis.^7^ Additionally, women treated with abaloparatide in ACTIVE experienced a decreased risk of vertebral, nonvertebral, and major osteoporotic fracture compared with placebo.^7^ Statistically significant changes in BTMs in women treated with abaloparatide were consistent with the observed changes in BMD.

The International Osteoporosis Foundation has indicated that the reference marker for bone formation should be PINP and CTX for bone resorption.^8^ Early changes in CTX and PINP are predictors of LS BMD response to teriparatide and risedronate treatment.^9^^,^^10^ Consequently, it has been proposed that BTMs can be used to monitor the effects of osteoporosis treatment using the least significant change approach.^11^

A previous study investigated the relationship between the changes in BTMs and subsequent LS BMD changes in women who participated in the ACTIVE study.^12^ An uncoupling index (UI), a mathematical approach for examining the balance between bone formation and resorption early in treatment and how it correlates with BMD increases was also calculated. A positive UI indicates that bone remodeling is unbalanced in favor of bone formation. A negative UI indicates an imbalance in favor of bone resorption. While calculation of UI may not be amenable for use in routine clinical practice, it can provide valuable information on the “anabolic window” for osteoporosis treatments and how this measure correlates with BTM and BMD changes.

In women from ACTIVE, early changes in PINP and the 3-mo UI correlated with the percentage change in LS BMD after 18 mo of treatment with abaloparatide.^12^ There have been no studies investigating the correlation between BTMs and changes in BMD in men undergoing treatment with anabolic agents for the treatment of osteoporosis to date.

This study compared the correlation of early BTM changes and subsequent increases in BMD at 12 mo between men treated with abaloparatide and those treated with placebo from the ATOM study. It compared the changes in the UI over time and compared men and women. The correlation between UI changes and subsequent 12-mo BMD changes was also compared between men treated with abaloparatide from the ATOM study and 12-mo results from women treated with abaloparatide in the ACTIVE study. We hypothesize that the correlation between early BTM changes and subsequent BMD changes with abaloparatide in men are similar to those reported for women.

## Materials and methods

### Study design and participants

The ATOM study (NCT03512262) was a randomized, double-blind, placebo-controlled, multicenter, phase 3 study and has been previously described.^6^ Briefly, men aged 40-85 yr with primary osteoporosis or osteoporosis with hypogonadism were randomized 2:1 to receive daily subcutaneous (SC) injections of abaloparatide 80 or placebo SC for 12 mo. Key inclusion criteria included BMD T-scores ≤−2.5 and >−3.5 at the LS, TH, or FN (based upon the male reference database) or ≤−1.5 and with radiologic vertebral fracture at screening or history of low-trauma nonvertebral fracture in the past 5 yr.

### Sample collection and BTM analyses

Blood samples were collected under fasting conditions at baseline and postdose at 1, 3, 6, and 12 mo and measured for PINP and CTX. Samples were batched and analyzed by Centre Académique de Recherche et d’Expérimentation en Santé (CARES) (University of Liège, Liège, Belgium). Assays for PINP were performed as a sandwich immunoassay using an IDS-iSYS autoanalyzer (ElectroChemiLuminescenceImmunoAssay or “ECLIA”). For repeatability, %CV were <5.5% and for reproducibility <6.2% for concentrations above the quantification limits. Assays for CTX were performed as a sandwich immunoassay using a COBAS E411 (Roche) autoanalyzer (ElectroChemiLuminescenceImmunoAssay or “ECLIA”).^6^ For repeatability, %CV were <4.6% and for reproducibility <5.4% for concentrations above the quantification limits.

### Statistical analyses

In this post hoc analysis, the correlation between change from baseline in concentrations of PINP and CTX at 1, 3, 6, and 12 mo was compared with percent change from baseline in LS BMD and TH BMD at 12 mo. Due to a skewed distribution of the BTM data, the correlation of the log ratio of PINP and CTX over baseline with percent change from baseline in LS BMD at 12 mo was evaluated using Pearson’s correlation coefficients. A *t* test was used to assess the difference in slopes of the regression lines between treatment groups. Linear regression analysis was performed to investigate the relationship between log ratio of PINP at 3 mo and LS BMD percent change at 12 mo for abaloparatide and placebo. For comparisons between men and women, Pearson correlation coefficients were assessed using the *z* score test after Fisher’s Z transformation. For women, 12-mo data from ACTIVE was used to match the 12-mo data from ATOM available for men even though ACTIVE generated data through 18 mo.

The UI was calculated as the *z* score of log PINP minus the *z* score of log CTX. The log transformed *z* score standardized marker values were calculated by taking the difference between each patient’s BTM and the mean of all patients’ BTMs at baseline divided by the associated standard deviation. All analyses were for exploratory purposes, and all tests were 2-sided and performed at a significance level of 0.05, with no adjustment for multiple comparisons. Analyses used observed data without imputing for missing values. The statistical software used was SAS version 9.4 (SAS Institute).

### Ethics

These studies (ACTIVE and ATOM) were conducted in accordance with the International Conference on Harmonization, the Declaration of Helsinki (2013), and applicable local regulations. Local institutional or central internal review boards (IRBs) were used to obtain approval from all institutions. All participants provided informed written consent to participate in the study.^6^

## Results

### Patient disposition and characteristics

In the ATOM study, a total of 228 men were randomized (abaloparatide, *n* = 149; placebo, *n* = 79). Of those randomized, 178 completed the study (114 [76.5%] in the abaloparatide group, and 64 [81.0%] in the placebo group). All 178 were included in the BTM analysis. Of the 2463 women who completed the ACTIVE study, 373 were included in the BTM analysis, 189 who received abaloparatide and 184 placebo.^12^ Baseline patient characteristics were similar between the abaloparatide and placebo groups in men from the ATOM study. Baseline characteristics were also similar between the ACTIVE and ATOM studies, except women had slightly higher baseline PINP and CTX values and a lower mean LS T-score than men (Table 1).

**Table 1:** Baseline characteristics of participants in BTM cohorts of ATOM and ACTIVE.

**Variable**	**ATOM**		**ACTIVE**	
	**Abaloparatide (*n* = 149)**	**Placebo (*n* = 79)**	**Abaloparatide (*n* = 189)**	**Placebo (*n* = 184)**
**Age (yr), mean (SD)**	68.5 (8.3)	67.8 (8.5)	68.6 (6.5)	68.4 (6.1)
**BMI (kg/m** ^**2**^**), mean (SD)**	26.6 (3.5)	26.5 (3.5)	25.0 (3.2)	24.9 (3.5)
**Race, *n* (%)** **^a^**				
**White**	140 (94.0)	76 (96.2)	154 (81.5)	148 (80.4)
**Asian**	8 (5.4)	1 (1.3)	28 (14.8)	33 (17.9)
**Black or African American**	0	1 (1.3)	6 (3.2)	3 (1.6)
**Other**	1 (0.7)	0	1 (0.5)	0
**Ethnicity, *n* (%)** **^b^**				
**Hispanic or Latino**	23 (15.4)	13 (16.5)	40 (21.2)	37 (20.1)
**Renal function, *n* (%)** **^c^**				
**Normal**	52 (34.9)	36 (45.6)	56 (29.6)	49 (26.6)
**Mild**	84 (56.4)	36 (45.6)	101 (53.4)	101 (54.9)
**Moderate**	13 (8.7)	7 (8.9)	32 (16.9)	34 (18.5)
**T-score, mean (SD)**				
**Femoral neck**	−2.1 (0.6)	−2.2 (0.7)	−2.1 (0.63)	−2.2 (0.65)
**Total hip**	−1.6 (0.7)	−1.7 (0.8)	−1.8 (0.77)	−1.9 (0.74)
**Lumbar spine**	−2.1 (1.1)	−2.1 (1.2)	−2.9 (0.88)	−2.9 (0.83)
**PINP, μg/L**				
**Median (IQR)**	48.2 (37.5, 61.3)	41.3 (33.8, 58.2)	50.6 (40.8, 65.3)	52.7 (40.9, 66.7)
**Mean (SD)**	50.0 (16.8)	47.0 (20.5)	54.5 (20.7)	56.6 (27.4)
**CTX, ng/L**				
**Median (IQR)**	327 (234, 475)	277 (210, 449)	476 (343, 588)	454 (338, 595)
**Mean (SD)**	360 (159)	336 (180)	486 (192)	488 (192)

a Classification based on physical or biologic characteristics.

b Classification based on shared culture, language, practices, and beliefs.

c Classification based on estimated creatinine clearance by Cockcroft-Gault formula.

Abbreviations: BTM, bone turnover marker; IQR, interquartile range; PINP, procollagen type I N propeptide.

### Correlation between BTMs and 12-mo BMD in men

**Figure 1:**
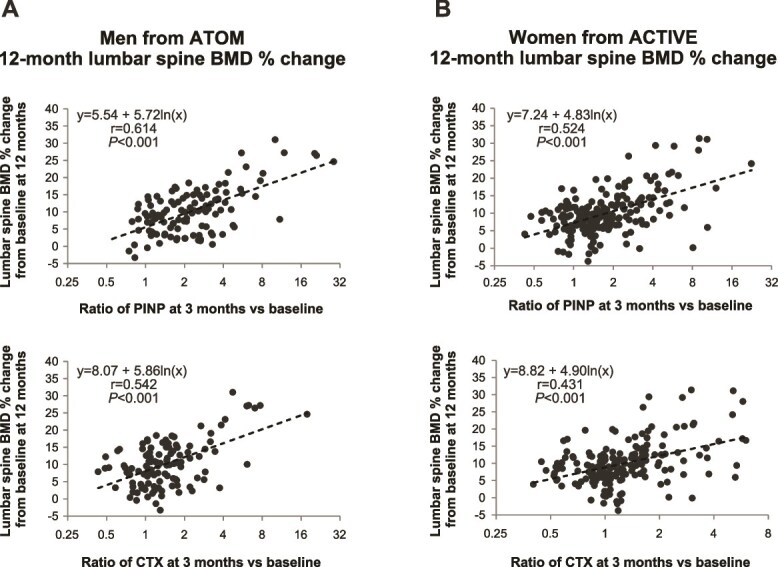
ATOM (A) and ACTIVE (B) scatter plot and linear regression of BTMs and LS BMD percent change in patients treated with abaloparatide. Abbreviations: BTM, bone turnover marker; PINP, procollagen type I N propeptide.

In men treated with abaloparatide, Pearson correlations for the log ratio of PINP over baseline and BMD percent change of LS from baseline at 12 mo were significant at 1 mo (*r* = 0.485, *p* < .0001), 3 mo (*r* = 0.614, *p* < .0001), 6 mo (*r* = 0.632, *p* < .0001), and 12 mo (*r* = 0.521, *p* < .0001) (Table 2, Figure 1). Pearson correlations for the log ratio of PINP over baseline and BMD percent change of TH at 12 mo was significant at 1 mo (*r* = 0.190, *p* = .040) but not 3, 6, or 12 mo in men treated with abaloparatide. There were no significant correlations observed in changes in PINP at any timepoint and LS or TH BMD at 12 mo in men treated with placebo.

**Table 2:** Correlation between log ratio of PINP over baseline and 12-mo BMD in men from ATOM.

		**LS**		**TH**	
		**Abaloparatide**	**Placebo**	**Abaloparatide**	**Placebo**
**1 mo**	*N*	117	65	117	65
	Corrcoef	**0.485**	−0.002	**0.190**	−0.204
	*p* value	**<.0001**	.9880	**.0398**	.1032
**3 mo**	*N*	114	61	114	61
	Corrcoef	**0.614**	0.078	0.184	−0.123
	*p* value	**<.0001**	.5479	.0504	.3441
**6 mo**	*N*	116	63	116	63
	Corrcoef	**0.632**	0.122	0.163	−0.289
	*p* value	**<.0001**	.3392	.0801	.0215
**12 mo**	*N*	118	65	118	65
	Corrcoef	**0.521**	0.079	0.051	−0.245
	*p* value	**<.0001**	.5320	.5832	.0495

Bold values represent significant changes. Abbreviations: Corrcoef, correlation coefficient; LS, lumbar spine; PINP, procollagen type I N propeptide; TH, total hip.

Pearson correlation results for the log ratio of CTX over baseline and percent change in LS BMD at 12 mo were significant at 1 mo (*r* = 0.353, *p* = .0001), 3 mo (*r* = 0.542, *p* < .0001), 6 mo (*r* = 0.526, *p* < .0001), and 12 mo (*r* = 0.413, *p* < .0001) in men treated with abaloparatide (Table 3; Figure 1). No significant correlations were observed for changes in CTX and 12-mo LS BMD in men treated with placebo. At the TH, there were no significant correlations between changes in CTX and 12-mo BMD for men treated with either abaloparatide or placebo.

**Table 3:** Correlation between log ratio of CTX over baseline and 12-mo BMD in men from ATOM.

		**LS**		**TH**	
		**Abaloparatide**	**Placebo**	**Abaloparatide**	**Placebo**
**1 mo**	*N*	117	64	117	64
	Corrcoef	**0.353**	−0.096	0.138	0.032
	*p* value	**.0001**	.4520	.1381	.8017
**3 mo**	*N*	114	60	114	60
	Corrcoef	**0.542**	−0.110	0.151	−0.011
	*p* value	**<.0001**	.4039	.1079	.9329
**6 mo**	*N*	116	63	116	63
	Corrcoef	**0.526**	−0.112	0.151	−0.022
	*p* value	**<.0001**	.3845	.1051	.8648
**12 mo**	*N*	118	65	118	65
	Corrcoef	**0.413**	−0.158	−0.055	−0.090
	*p* value	**<.0001**	.2101	.5516	.4761

Bold values represent significant changes. Abbreviations: Corrcoef, correlation coefficient; LS, lumbar spine; TH, total hip.

### Three-month BTMs and Twelve-month LS BMD in men compared to women

The relationship between the log ratio of PINP at 3 mo and percent change in LS BMD at 12 mo was significant in women treated with abaloparatide (*r* = 0.524, *p* < .0001). The correlation coefficient results were similar between men and women (*p* = .267). The relationship between early increase in CTX and increases in LS BMD in women treated with abaloparatide was also significant (*r* = 0.431, *p* < .0001) and consistent with results in men (*p =* .227 for men vs women). In ATOM, mean (SD) LS BMD increase was 11.4% (±6.8) in men with PINP increases greater than 10 μg/L (*n* = 89) and 6.2% (±4.8) in men with PINP increases less than 10 μg/L (*n* = 25) (*P* < 0.05). In ACTIVE, mean (SD) LS BMD increase was 10.9% (±6.6) in women with PINP increases greater than 10 μg/L (*n* = 132) and 7.8% (±4.0) in women with PINP increases less than 10 μg/L (*n* = 54) (*p* < 0.05).

### Uncoupling index

Median PINP levels were comparable in abaloparatide-treated men from ATOM and abaloparatide-treated women from ACTIVE at baseline and 1, 3, 6, and 12 mo (Table 4). Median CTX was significantly higher in women from ACTIVE at baseline (476 vs 327 ng/L, *p* ˂ .0001), month 1 (462 vs 333 ng/L, *p* ˂ .0001), and month 3 (567 vs 421 ng/L, *p* = .0019) compared to men from ATOM. Significant differences were not seen at months 6 and 12.

**Table 4:** Bone turnover markers and uncoupling index in men from ATOM compared to women from ACTIVE.

	**ATOM (*n* = 149)**	**ACTIVE (*n* = 189)**	***p* value**
**Median (IQR) PINP, μg/L**			
**Baseline**	48.2 (37.5, 61.3)	50.6 (40.8, 65.3)	.0672^a^
**1 mo**	109.0 (70.9, 164.5)	100.5 (71.3, 137.6)	.1087^a^
**3 mo**	89.2 (59.7, 145.7)	87.7 (53.9, 134.5)	.3546^a^
**6 mo**	91.2 (57.7, 182.4)	86.7 (51.1, 140.7)	.2579^a^
**12 mo**	85.7 (56.1, 180.7)	82.6 (50.7, 133.1)	.1615^a^
**Median (IQR) CTX, ng/L**			
**Baseline**	327 (234, 475)	476 (343, 588)	<.0001^a^
**1 mo**	333 (234, 442)	462 (305, 610)	<.0001^a^
**3 mo**	421 (291, 648)	567 (369, 936)	.0019^a^
**6 mo**	476 (284, 744)	536 (309, 852)	.4513^a^
**12 mo**	448 (273, 701)	494 (280, 796)	.3698^a^
**Mean (SD) UI**			
**1 mo**	2.26 (1.62)	1.74 (1.20)	.001^b^
**3 mo**	1.32 (1.24)	0.88 (1.05)	.0008^b^
**6 mo**	1.36 (1.29)	1.25 (0.84)	.4238^b^
**12 mo**	1.34 (1.23)	1.15 (0.91)	.1464^b^

a *p* value calculated using Wilcoxon rank sum test.

b *p* value calculated using *z* test.

Abbreviations: IQR, interquartile range; PINP, procollagen type I N propeptide; UI, uncoupling index. Reproduced from *J Bone Miner Res* 2024;39(Supplement 1):i1-i406, with permission of the American Society for Bone and Mineral Research. © American Society for Bone and Mineral Research, 2025.

**Figure 2:**
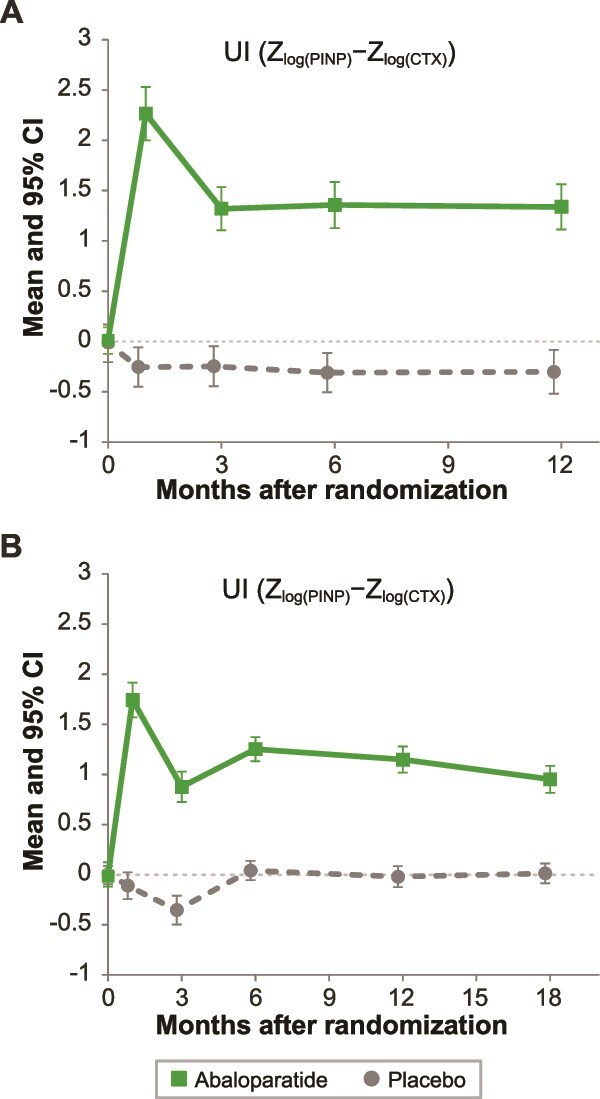
Mean (95% CI) of uncoupling index (UI) in (A) men from ATOM and (B) women from ACTIVE.

The UI was higher for participants treated with abaloparatide compared to placebo over time for both men and women (Figure 2). UI was greater in men compared to women at month 1 (2.26 vs 1.74, *p* = .001) and month 3 (1.32 vs 0.88, *p* = .0008) (Table 4). The mean (SD) log *z* score at 3 mo was higher for men than for women for both CTX (men 0.80 [1.44], women 0.57 [1.65]) and PINP (men 2.12 [2.05], women 1.45 [1.82]), with a bigger difference for PINP. The UI was not significantly different at 6 mo (men, 1.36; women, 1.25; *p* = .4238) and 12 mo (men, 1.34; women, 1.15; *p* = .1464).

There was a significant positive correlation between the UI as early as 1 and 12 mo LS BMD in men treated with abaloparatide (*r* = 0.362; *p* < .001, Table 5). The correlation increased at month 3 (*r* = 0.453, *p* < .0001), month 6 (*r* = 0.558, *p* < .0001), and month 12 (*r* = 0.474, *p* < .0001). In women, the correlation between UI and 12-mo LS BMD was significant at 3 mo (*r* = 0.252, *p* = .0005), 6 mo (*r* = 0.293, *p* < .0001), and 12 mo (*r* = 0.262, *p* = .0003). The correlation between UI and 12-mo LS BMD was stronger in men compared to women at 1, 6, and 12 mo (*p* < .05).

**Table 5:** Correlation between UI and LS BMD at 12 mo in men from ATOM and women from ACTIVE.

	**ATOM (*n* = 149)**	**ACTIVE (*n* = 189)**	***p* value ATOM vs ACTIVE**
**UI at 1 mo**			.0266
**Correlation coefficient (*r*)**	0.362	0.114	
***p* value**	<.0001	.1213	
**UI at 3 mo**			.054
**Correlation coefficient (*r*)**	0.453	0.252	
***p* value**	<.0001	.0005	
**UI at 6 mo**			.0060
**Correlation coefficient (*r*)**	0.558	0.293	
***p* value**	<.0001	<.0001	
**UI at 12 mo**			.0374
**Correlation coefficient (*r*)**	0.474	0.262	
***p* value**	<.0001	.0003	

Abbreviation: UI, uncoupling index.

## Discussion

There is a lack of data on the relationship between BTMs and BMD in men undergoing treatment for osteoporosis, particularly in those receiving an anabolic agent. This study is the first to analyze this relationship in men treated with abaloparatide. A clear correlation was seen between early increases in PINP and CTX and subsequent increases in LS BMD at 12 mo. These results were stronger than BTM and BMD correlations seen in women at 12 mo, highlighting a potential effect of gender on these correlations.

There are two possible explanations for the stronger correlations between BTM and change in BMD in men than women. It could be that the BTM data in men showed less variance than the BTM data in women, which could be reflective of a more homogenous study population in ATOM based on inclusion criteria. Another explanation might be that the greater increase in BTM is the cause of the stronger *r* values, as the change in the predictor variable has a positive effect on the correlation coefficient.

Another issue to explain is why changes in BTM correlate more strongly with changes in BMD at the LS than the TH. In the ATOM study, the change in LS BMD at 12 mo was more than three times greater than the change in TH BMD,^6^ and correlations are very sensitive to the magnitude of change in the covariates.

The correlation between BTMs (PINP and CTX) and BMD has been previously analyzed in women and men treated with other osteoporosis medications.^13^^–^^17^ Eastell et al showed that 6-mo changes in PINP and CTX were moderately correlated with LS or TH BMD changes in women after 36 mo of denosumab treatment.^16^ Tsujimoto et al.^17^ showed a strong relationship between early change in PINP and later change in LS BMD during teriparatide therapy in women and men; however, there were only 14 men included in the study. A meta-analysis of studies that examined the relationship between BTMs and BMD showed a correlation of baseline BTMs with long-term BMD changes from baseline after drug intervention.^13^ However, the strength of the correlations varied depending on bone site, intervention, and time duration of BTM measurements. Correlations were strongest with PINP and urine/serum CTX. Correlations with anabolics were inconsistent in the meta-analysis, highlighting the need for investigations, like the current study, that focus on anabolics.

Previously, UI has been used to assess the balance between bone formation and resorption early in treatment and has been suggested to be more predictive of subsequent changes in BMD than individual measures of BTMs.^12^^,^^18^ Lane et al.^18^ reported significant correlations between UI at 1, 3, and 6 mo and 12 mo changes in spine BMD in postmenopausal women treated with parathyroid hormone fragment. Eastell et al.^12^ found a significant correlation between 3-mo UI and LS BMD after 18 mo of abaloparatide treatment in women. In our study, UI in men peaked at 1 mo, remained positive through 12 mo, and was significantly correlated with 12-mo LS BMD at all time points. Although the UI values in men were higher than those found in women, it should be noted that the data for men and women are not from the same study and were not designed to be compared directly. We speculate that the magnitude of the increase in UI is greater in men due to the baseline bone turnover (especially CTX) being lower in men than women, yet the levels of treatment are similar. The shared pattern of an early peak in BTMs and UI, which remained elevated during treatment, coupled with significant correlation to changes in LS BMD, suggest that BTM and BMD correlations are similar or stronger in men compared to women.

The greater increase in UI in men as compared to women does not imply that the BMD increase would be expected to be higher in men than women. Indeed, the percent increases at 12 mo in the ACTIVE and ATOM trials were similar.^6^^,^^7^ It is likely that it is the absolute change in BTMs that relate more closely to the BMD increase than the relative change, and women have a higher baseline BTM, particularly CTX.

The current study has a practical implication for clinical practice. Some authorities recommend monitoring anabolic treatment response by measuring PINP at 1 and 3 mo after treatment initiation.^11^^,^^19^ It has been proposed that when we monitor anabolic therapies, an increase in PINP beyond the least significant change indicates a positive response.^11^ A 10 μg/L increase in PINP has previously been suggested as a cutoff for the least significant change for predicting clinical improvement in LS BMD.^19^ For teriparatide, mean (+SD) LS BMD increases at 12 mo were 9.5 ± 6.2% (*n* = 172) in patients with PINP increases greater than 10 μg/L, and 7.6 ± 5.5% (*n* = 55) for patients with PINP increases less than 10 μg/L (*p* < .05).^20^ Similarly, with abaloparatide, mean LS BMD increases were 10.9 (±6.6%) (*n* = 132) and 7.8 (±4.0%) (*n* = 54) (*p* < .05) in ACTIVE and 11.4 (±6.8%) (*n* = 89) and 6.2 (±4.8%) (*n* = 25) (*p* < .05) in ATOM for patients with PINP increases greater than or less than 10 μg/L, respectively. The mean BMD difference between cohorts with and without 10 μg/L increases in PINP is slightly wider in those with greater increases, suggesting 10 μg/L or greater increase in PINP as an indicator of clinical meaningful LS BMD gains at month 12. This study provides evidence that an increase in PINP in men treated with abaloparatide likely translates into a BMD response at 1 yr and so helps build a case for monitoring individual patients where early efficacy data is required. If PINP increases are less than the least significant change, injection technique, medication adherence, and the possibility of secondary osteoporosis should be assessed.

Strengths of this study include the large sample size given that surrogates are being studied, the continuous outcomes, and the adjustments taken when analyzing the data to account for the skewed distribution of BTMs. Although ATOM and ACTIVE were not explicitly created to be directly compared, the designs of the studies were very similar and our finding of strong parallels in the BTM-BMD responses in men and women is convincing. The data provide further evidence that men respond to osteoporosis treatment similarly to women, compatible with a recent observational study.^20^ The use of log-transformed BMD data for these analyses and lack of fracture data limits the clinical application of the current data.

In conclusion, a significant correlation was seen between levels of BTMs early in the course of treatment and subsequent increases in BMD in men from the ATOM study. The correlation between early changes in PINP and percentage change in LS BMD after 12 mo of treatment with abaloparatide is similar in men and women, with a slightly higher log ratio in men versus women. Similar correlations were found in UI for men and women, with a slightly higher UI in men. These data support that early changes in BTMs may reflect subsequent changes in BMD in both men and women treated with abaloparatide. Future work is needed to standardize and understand the applicability of utilizing early changes in BMD to monitor treatment response in patients with osteoporosis. In addition, the immediacy of beneficial effects of therapy can impact adherence to treatment;^21^ therefore, the ability to monitor early impact of anabolic therapy on bone could potentially be beneficial to encourage patient compliance.

## Supplementary Material

ff3aca0a-0d9e-4beb-9a3e-2ab19a350fac_zjaf003

## Data Availability

Data that underlie the results reported in a published article may be requested for further research 6 months after completion of FDA or EMA regulatory review of a marketing application (if applicable) or 18 months after trial completion (whichever is latest). Radius will review requests individually to determine whether (1) the requests are legitimate and relevant and meet sound scientific research principles and (2) are within the scope of the participants’ informed consent. Prior to making data available, requestors will be required to agree in writing to certain obligations, including without limitation, compliance with applicable privacy and other laws and regulations. Proposals should be directed to info@radiuspharm.com.
